# New insights into regulatory cell death and acute pancreatitis

**DOI:** 10.1016/j.heliyon.2023.e18036

**Published:** 2023-07-07

**Authors:** Hongyao Li, Ding Wu, Haidan Zhang, Peiwu Li

**Affiliations:** Department of Emergency, Lanzhou University Second Hospital, Lanzhou University, Lanzhou, Gansu, 730030, China

**Keywords:** Acute pancreatitis, RCD, Autophagy, Necroptosis, Pyroptosis, Ferroptosis

## Abstract

Acute pancreatitis (AP) may be associated with both local and systemic complications. Although it is usually self-limiting, up to 20% of patients develop severe acute pancreatitis (SAP), which leads to systemic inflammatory response syndrome (SIRS) and multiorgan dysfunction and failure affecting the lung, kidney, liver and heart. Patients who survive the condition frequently develop devastating long-term consequences such as diabetes mellitus, exocrine pancreatic insufficiency, chronic pancreatitis (CP) and poor quality of life. A lack of specific targeted treatments is the main reason for high mortality and morbidity, indicating that more research on the pathogenesis of AP is needed. In the past decade, substantial advancements have been made in our understanding of the pathophysiological mechanisms of AP, including mechanisms of calcium-mediated acinar cell injury and death, the cytoprotective role of the unfolded protein response (UPR) and autophagy in preventing sustained endoplasmic reticulum stress (ERs); however, the mechanism of parenchymal cell death is relatively poorly understood. This paper reviews the research progress of the regulatory cell death (RCD) mode in the pathogenesis of AP, providing some new insights and regulatory targets for the pathogenesis and treatment of AP, facilitating better targeted drug development.

## 1 Introduction

Acute pancreatitis (AP) is an inflammatory disease of the pancreas. The prognosis depends mainly on the development of organ failure and secondary infection with pancreatic or peripancreatic necrosis. Gallstones and alcohol abuse are the two most common causes of AP. Other causes include medication, endoscopic retrograde cholangiopancreatography (ERCP), hypercalcemia, and hypertriglyceridemia. With the significant increase in obesity in recent years, hypertriglyceridemia has become the third leading cause of AP [1]. AP is usually a mild, self-limiting disease, but nearly 20% of people with AP develop severe acute pancreatitis (SAP), which can lead to systemic inflammatory response syndrome (SIRS) and multiple organ dysfunction and failure (MODS), with mortality ranging from 8% to 39%. The main causes of death in SAP patients are intestinal barrier dysfunction (IBFD), acute lung injury (ALI), acute renal failure (AKI) [2]. Over the past 10 years, the treatment of acute pancreatitis has moved toward multidisciplinary, tailored and minimally invasive treatment. Despite significant improvements in the treatment and management of patients with SAP, the mortality rate remains high. Therefore, it is very important to study the pathogenesis of AP, especially SAP [1,2].

## 2 Pathophysiology

AP is characterized by impairment of pancreatic acinar cells (functional units of pancreatic exocrine secretion), which promotes inappropriate release of trypsinogen to trypsin acinar cells, triggering the activation of other digestive enzymes, the kinin system and the complement cascade, which leads to automatic digestion of the pancreatic parenchyma [3]. Trypsin will not only digest the organ itself but also the blood vessel wall and the pancreas adjacent to the gastrointestinal tract, leading to its perforation. The self-digestion of organs leads to very strong local or systemic inflammation, which may lead to multiple organ failure (MOF), similar to sepsis [4]. Blocked ducta (e.g., gallstone pancreatitis) is one of the most common causes of pancreatic injury, which can lead to increased pancreatic duct pressure, interstitial edema and accumulation of fluid rich in enzymes in pancreatic tissue [3,4]. The pathogenesis of AP includes pathological calcium signal transduction, mitochondrial dysfunction, premature activation of trypsinogen in acinar cells and macrophages, endoplasmic reticulum stress (ERs), unfolded protein reaction (UPR), and autophagy impairment. The death of acinar cells can trigger a strong aseptic inflammatory reaction, and this process can lead to pancreatic injury [5]. Studies have shown that dead and injured pancreatic acinar cells can release damage-related molecular patterns (DAMPs), such as histones, DNA and heat shock proteins (HSPs). The accumulation of DAMPs with proinflammatory effects, the subsequent activation of inflammatory bodies and the release of inflammatory factors may aggravate pancreatic injury and lead to SIRS and MOF [6].The systemic complications and disease severity of AP largely depend on the intensity of the immune response. AP starts from the early activation of digestive enzymes in pancreatic acinar cells, leading to cell damage. Parallel to this process is the infiltration of inflammatory cells, in which macrophages and neutrophils first reach the organs and cause pancreatic injury [7]. During SAP, macrophages (the main leukocyte population infiltrating the pancreas) have been shown to differentiate into the proinflammatory M1 phenotype and phagocytose necrotic tissue regions. The initial immune response is characterized by the release of proinflammatory cytokines, which can lead to SIRS [8]. This stage of hyperinflammation is usually accompanied by compensatory anti-inflammatory response syndrome (CARS), which is related to immunosuppression and may promote secondary pancreatic necrosis. The intensity and order of SIRS and CARS are the key prognostic factors of AP. Excessive inflammation can lead to shock and may lead to multiple organ dysfunction syndrome (MODS), which is associated with high mortality in the early stage of the disease. On the other hand, immunosuppression during CARS allows for bacterial translocation into pancreatic necrosis or severe sepsis and is related to an increase in mortality in the later stages of the disease [9,10].

## 3 Regulated cell death and acinar cells

Cell death plays a central role in all aspects of life and participates in the development and tissue homeostasis of multicellular organisms. Therefore, there is increasing interested in treating diseases by influencing RCD. With the discovery of the mechanisms of different death modes, the entire RCD network has expanded, and the relationship between RCDs has become complex [11]. RCD occurs through different subprograms, leading to cell decomposition in different ways, with different morphological changes and immune consequences [12]. RCD not only plays a role in maintaining the internal environment of the body but also provides a strong signal to stimulate local inflammation or the systemic immune response by releasing DAMPs. Significant progress has been made in revealing the molecular mechanism of the inflammatory response. In AP, little is known about the pathway of parenchymal cell death. Recent studies have found that RCD of acinar cells includes mainly apoptosis, autophagy, pyroptosis, necroptosis and ferroptosis [13,14].

### 3.1 Apoptosis (Fig. 1)

Apoptosis is the earliest described form of RCD and plays a key role in tissue homeostasis. It contributes to cell turnover, normal function of the immune system and embryonic development [11]. Apoptosis includes both external and internal pathways. The first pathway needs to be initiated by activating death receptors (such as TNF receptors or Fas receptors) and is mediated by caspase-8, which is activated by various micro environment disturbances. Conversely, intrinsic apoptosis is ignited by mitochondrial outer membrane permeabilization (MOMP), which leads to the release of mitochondrial proteins (e.g., cytochrome C, somatic (CYCS), diablo IAP-binding mitochondrial protein (DIABLO, also known as Smac), and HtrA serine peptidase 2 (HTRA2)) and subsequent activation of initiator caspase CASP9 [12]. Some morphological changes in apoptotic cells have been fully identified and recorded. These changes include cell blistering and contraction, nuclear fragmentation, coagulation and fragmentation of genetic material (chromatin and nucleosome DNA), and the formation of vesicles called apoptotic bodies (ApoBDs). This last process is called efferocytosis. Although the traditional view holds that efferocytosis is the end point of apoptosis and that it ends the life of diseased cells, increasing evidence focuses on the transfer, recycling and even reuse of material packaging in ApoBD [15].

**Fig. 1:**
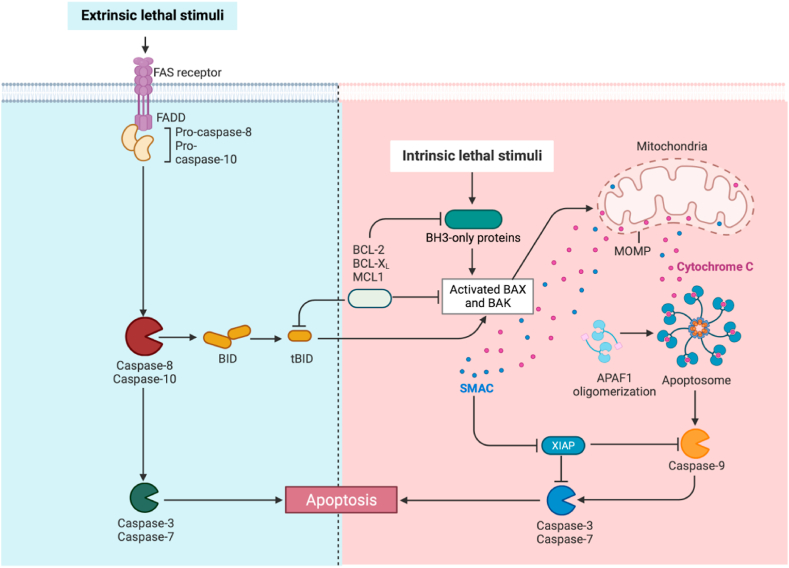
Apoptosis include extrinsic and intrinsic pathways. The first pathway needs to be initiated by activation of death receptors, such as TNF receptors or Fas receptors, and mediated by caspase-8, which is initiated by various microenvironmental perturbations. Intrinsic apoptosis is ignited by mitochondrial outer membrane permeabilization (MOMP) that leads to the release of the mitochondrial proteins (e.g., cytochrome C, somatic (CYCS), diablo IAP-binding mitochondrial protein (DIABLO, also known as Smac), and HtrA serine peptidase 2 (HTRA2) and subsequent activation of initiator caspase CASP9 [8,13].

In the animal model of AP, approximately 1%–5% of acinar cells are apoptotic. Careful biochemical and morphological examination of the experimental AP model shows that SAP (in mice) mainly involves necrosis and rarely apoptosis [16,17]. Cerulein, l-arginine, ethanol, lipopolysaccharide (LPS), platelet activating factor and cytokine (e.g., tumor necrosis factor (TNF-α) and interleukin (IL-1β) have been shown to induce apoptosis of AP cells. In addition to caspases and Bcl-2 family proteins, other components of the death mechanism (such as X-linked apoptosis inhibitors and p53) also participate in the regulation of external or internal apoptosis in AP [18,19]. In addition, the pancreas is particularly vulnerable to endoplasmic reticulum stress (ERs) because acinar cells produce many proteins; this stress usually occurs in acinar cells in AP. Common pancreatic toxins (e.g., alcohol and its metabolites) increase the amount of protein (e. g. trypsinogen, chymotrypsinogen, lipase and lysosomal histone B). By reducing the ability of cells to process and recover unwanted proteins (i.e., dysfunctional autophagy and mitochondrial dysfunction), if ER persists, it will induce apoptosis [5,20,21]. Although the UPR attempts to limit further protein accumulation through translation weakening to protect cells, transcriptional activation of genes encoding ER resident partners can increase ER folding capacity and induce ER-related degradation to remove misfolded molecules. However, if these mechanisms are not sufficient, the stress-damaged cells can be eliminated by completely inducing apoptosis [[22], [23], [24]]. Studies have shown that p53-AIFM 2-ATF 6 is highly expressed in pancreatic tissues of SAP patients and PRSS 1 Tg mice and can regulate apoptosis in acinar cells. Activated transcription factor 6 (ATF 6) is a transmembrane glycoprotein on the ER membrane that not only acts as a UPR sensor/converter but also acts as a transcription factor. When misfolded proteins accumulate in the ER, ATF 6 is transferred to the Golgi apparatus, where it is processed to produce cytoplasmic fragments, which are the main medium for the adaptive response of ER protein misfolding [25]. In recent years, a large number of studies have shown that some phytoceuticals, such as vincristine [26], colchicine [27], resveratrol [4] and nimblet [28] can alleviate AP by regulating acinar cell apoptosis and inhibiting the cellular inflammatory response. However, some researchers have also observed that apoptotic pancreatic follicular cells can release histones, DNA, and high mobility histone B1 (HMGB1) to promote pancreatic injury and inflammation. Whether the induction or inhibition of apoptosis is beneficial in the clinical environment remains to be confirmed [8,20].

### 3.2 Autophagy (Fig. 2)

Autophagy is defined as a conservative catabolic process in all eukaryotes. Its main role is to degrade the content of the cytoplasm, restore damaged organs and proteins, and maintain the homeostasis of cells when cells face hunger and other stress factors [29]. Autophagy is critical to the response to various stressors (i.e., hypoxia, infection, ERs, tissue remodeling). The current classification of autophagy can be divided into three categories: macroautophagy, microautophagy and chaperone-mediated autophagy (CMA) [21].

**Fig. 2:**
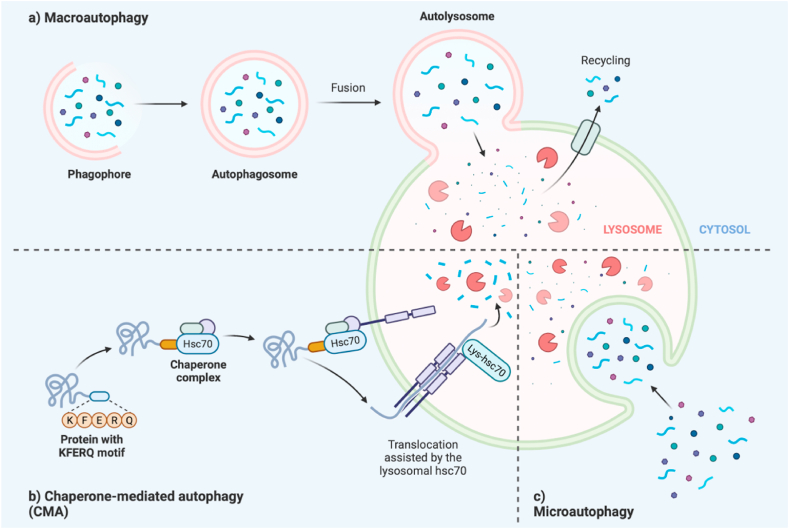
Autophagy is mainly divided into macroautophagy, microautophagy and chaperone mediated autophagy (CMA). CMA has strong specificity and is important for the activation of pancreatic enzymes [4].

Impaired autophagy is characteristic of various experimental acute pancreatitis models.

Autophagy occurs at a basic rate in most cells as a quality control mechanism to eliminate protein aggregates and damaged or unwanted organelles [30]. However, impaired autophagy leads to trypsinogen activation and ER and mitochondrial dysfunction, making acinar cells more vulnerable to injury and death [7]. The basic autophagy rate of the exocrine pancreas in mice is higher than that of the liver, kidney, heart or endocrine pancreas because the exocrine pancreas has the characteristic of a high protein synthesis rate [31]. Research shows that autophagy activates trypsinogen by delivering trypsinogen to lysosomes, which may damage acinar cells during AP attack. On the other hand, Marerinova et al. [32] showed that delayed autophagy was related to the imbalance between histone L (degradation of trypsinogen and trypsin) and histone B (conversion of trypsinogen to trypsin), leading to the accumulation of active trypsin in AP in acinar cells. Autophagy also leads to mitochondrial and ER dysfunction and lipid metabolism disorder, which further increases the severity of AP [24]. In mice whose pancreas specifically knocks out key mediators of autophagy formation (such as autophagy-related protein (ATG) 5 and ATG7), spontaneous pancreatitis occurs in mice, characterized by trypsinogen activation, fibrosis, inflammation, acinar cell to duct edema, and pancreatic atrophy. The application of alginate (a natural disaccharide known to enhance autophagy) largely prevents trypsinogen activation and pancreatic necrosis [31,33]. The completion of autophagy and the degradation of goods require the interaction between autophagosomes and lysosomes. Lysosome-associated membrane protein (LAMP) is an important regulator of lysosomal function and biogenesis [3]. It has been reported that the loss of LAMP1 and LAMP2 during acute pancreatitis is the mechanism leading to the interruption of autophagic flow, vacuolization and AP progression. The deletion of transcription factor EB (TFEB) is another mechanism to reduce autophagy in acute pancreatitis. TFEB is a major regulator of lysosomal biogenesis and induces the transcription of autophagy-related genes. In the peptide model of AP, the rapid degradation of TFEB in rain frogs leads to autophagy defects. In addition, decreased cytoplasmic TFEB is associated with human pancreatitis [34]. Moreover, mitochondria and autophagy are the most studied selective autophagy modes in AP. In mitochondrial phagocytosis, the selective degradation of mitochondria removes dysfunctional organelles, reduces ROS levels, prevents lipid peroxidation, and reduces the efficiency of iron prolapse. To date, more than 10 kinds of receptors involved in mitosis have been identified, including chelator 1 (SQSTM1), optic nerve protein (OPTN), oiled coil domain 2 (CALCOCO2) and Tax1 binding protein 1 (TAX1BP1) [35,36]. Intestinal mucosal barrier damage can lead to intestinal flora translocation, leading to secondary infection of pancreatic tissue. Autophagy also plays an important role in SAP-induced intestinal injury. Current research shows that autophagy can maintain intestinal homeostasis by 1) eliminating invading microorganisms and toxins; 2) protecting the tight junctions (TJs) and gap junctions (GJs) of the intestine from vesicle turnover; 3) maintaining the secretion of Paneth cells (PCs) and goblet cells; 4) balancing the intestinal immune response; 5) regulating ROS and inflammation; 6) producing antifibrotic effects; and 7) balancing the regeneration of intestinal epithelial cells (ISCs) [37]. Chloroquine (CQ) and its derivatives have been widely used to inhibit autophagy in vitro, with relatively low toxicity. CQ administration significantly delayed colon shortening, inflammatory cell infiltration, tissue damage and weight loss [38]. Similarly, it has been reported that glutamine limits stress-induced apoptosis by regulating mammalian targets of rapamycin (mTOR) and the mitogen-activated protein kinase/p38 pathway, thereby enhancing autophagy of IECs under basic and stress-induced conditions [39]. In addition, recent studies have shown that bone marrow mesenchymal stem cells inhibit autophagy in multiple organs including the pancreas, small intestine and lung to prevent multiple organ damage caused by SAP [40].

### 3.3 Necroptosis (Fig. 3)

The regulatory process of necrosis is called necroptosis, which is a regulated, proinflammatory, and caspase-independent form of cell death. During AP, necroptosis is mediated by receptor interacting protein kinases (RIPs), including RIP1-RIP3 and mixed lineage kinase domain-like protein (MLKL) pathways, where MLKL is phosphorylated by RIP3, leading to its oligomerization. The MLKL oligomer then transfers to the plasma membrane, eventually leading to plasma membrane perforation and overflow of the cell contents. Compared with apoptosis, necroptosis may be a more aggressive mode of cell death. Recent studies have shown that necroptosis may be the main mechanism of acinar cell death in AP. [5,11],

**Fig. 3:**
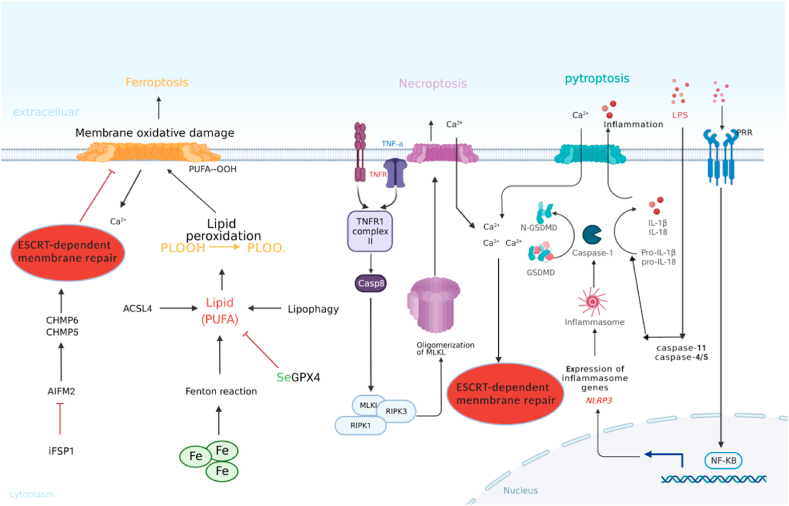
Necroptosis, pyroptosis and ferroptosis in acute pancreatitis [39,41].

Studies have shown that 40% of cells undergo necroptosis in pancreatitis, which is characterized by the formation of necrotic vesicles; RIP3 deletion or necrostatin treatment can improve pancreatitis. In addition, necroptosis releases DAMPs, which activate the pathway containing the protein 3 (NLRP3) domain of the NLR family, leading to focal ptosis [17]. Caerulein-induced acinar cell necroptosis depends on an increase in cytoplasmic calcium levels before pathology and occurs after ATP depletion in acinar cells. In addition, MLKL deficiency or RIP3 deficiency plays a protective role in caerulein-induced AP [43,44]. Some studies have shown that in AP, the expression levels of RIPK3 and phosphorylated MLKL are positively correlated with the degree of necroptosis, while the expression levels of RIPK1 are negatively correlated with the degree of necroptosis. Knocking down RIPK1 can inhibit NF-κB activation and promote acinar cell necrosis. Therefore, overexpression of RIPK1 inhibits the inhibition of RIPK3, reduces the phosphorylation level of MLKL, and reduces the necroptosis of acinar cells, providing protection in AP mice [45]. In addition, necroptosis plays a key role in SAP-related intestinal injury and SAP-related lung injury (ALI). Nec-1 or RIP3 knockout can significantly reduce organ damage [46]. However, other studies have shown that RIP 3- and MLKL-mediated necroptosis plays a protective role in AP. Pancreatic edema and inflammation of RIPK3 ^−/−^ and MLKL ^−/−^ mice are more serious and more inflammatory cells are recruited into the pancreas compared to control groups. In addition, the lack of MLKL leads to the downregulation of antiapoptotic genes, thereby increasing the rate of apoptosis [47]. Previous studies on pancreatitis and necrosis have reported conflicting results, which may be attributed to differing induction drugs, treatment durations and necrosis observation time windows. However, necroptosis is undoubtedly closely related to the progression of pancreatitis and further research is needed to confirm the relationship between them [45]. In terms of treatment, serum amyloid A (SAA) is a sensitive biomarker in the early stage of inflammatory diseases and studies have shown that SAA3 induces the RIPK3-dependent necroptosis pathway and leads to AP in acinar cells, which suggests that this pathway may be a potential drug target of AP [48]. MiR-21 and miR-325-3 can slow the development of AP by affecting the necroptosis RIPK3/MLKL signaling pathway in AP [8,20]. Bone marrow mesenchymal stem cells (BMSCs) can inhibit cytokines (such as IL-1β, IL-6 and TNF-α), increase the level of anti-inflammatory mediators (such as IL-4 and IL-10) and regulate the RIPK1/RIPK3/MLKL-mediated necroptosis pathway to reduce the severity of SAP, thereby reducing the systemic inflammatory response [41]. CaMKII is a calcium regulatory protein that is upregulated in pancreatic necroptosis of AP mice. The inhibitor KN93 protects mice from AP by reducing oxidative stress products, reducing the expression levels of the RIPK3 and *p*-MLKL pathways and preventing necroptosis [45,46]. The application of Nec-1, a necrosis inhibitor, before active fluid resuscitation can reduce the serum and cytoplasmic levels of HMGB 1 (which can determine intestinal barrier dysfunction and infection in SAP patients), which may reduce inflammation of SAP-related organs in mice [49].

### 3.4 Pyroptosis (Fig. 3)

Pyroptosis is an inflammatory process of RCD and a functional product of NLRP3 and other inflammasomes regulated by PRRs. Caspase-1, caspase-4, caspase-5 and caspase-11 directly cleave the substrate gasdermin D (GSDMD) to produce N-terminal fragments. GSDMD mediates the formation of pores in the plasma membrane, followed by cell swelling and membrane rupture, releasing a large amount of cytoplasmic content [6]. The activation of the pyroptosis pathway mainly includes the caspase-1-dependent canonical pathway and the caspase-4/5/11-dependent non-canonical pathway. Later studies also showed that caspase-3, caspase-7 and caspase-8, which play a role in apoptosis, also participate in the regulation of pyroptosis [50].

Research shows that damaged acinar cells are characterized by pyroptosis. When injury occurs, the rupture or death of acinar cells or the release of damaged cell contents induces the activation of DAMPs, Toll-like receptor 4 (TLR4) and TLR9 in immune cells [42], and protein 1 (NOD1) containing the nucleotide binding oligomeric domain in pancreatic follicular cells. These pathways induce NF-κB activation, the NLRP3 inflammasome and effectors (IL-1β, IL-18 and HMGB1), which can trigger aseptic inflammatory reactions in the pancreas and play a role in magnifying inflammatory reactions [6,31]. When AP further progresses to SAP, caspase-1, IL-18 and IL-1β in acinar cells are mediated by TLR4 and damage-associated molecular patterns (DAMPs) [51]. At the same time, downstream inflammatory factors released by pyroptosis also play important roles in the inflammatory transmission of AP. HMGB1 is the main endogenous ligand of TLR4 in AP. HMGB1 released by immune cells (mainly leukocytes) is regulated by caspase-1 activation and coordinates the inflammatory response in sepsis or early AP. When HMGB1 is released in acinar cells, it may lead to pyroptosis and immunosuppression, thereby weakening the host's ability to eliminate microbial infection [52,53]. IL-1β has been proven to play a mediating role in promoting local tissue destruction and distant organ complications, and IL-1β gene defects or injection of IL-1β antagonists can reduce the injury reactions of experimental AP patients [54]. IL-1β also activates antigens, and induces CD4^+^ T cells to differentiate into Th1 and Th17 cells. In addition to caspase-1, GSDMD is also strongly involved in IL-1 through the formation of GSDMD pores and membrane disruption [55]. The role of IL-18 in AP seems more complex. IL-18 is crucial for intestinal homeostasis, especially for intestinal epithelial host defense, because its direct bactericide increases the stability of intestinal mucosal barrier function [56]. IL-18, also known as interferon- γ inducer, a new type of proinflammatory cytokine, is associated with IL-1β in structure and function. IL-18 induces IL-1 β and TNF-α gene expression and synthesis, as well as some chemotaxis. In addition, IL-18 plays a role in the Th-1 response to viral antigen stimulation because IL-18 induces natural killer cells and produces interferon γ T cells. The level of IL-18 in SAP patients is significantly increased. The occurrence of pancreatic pyroptosis and organ failure is closely related to the concentration of IL-18 in serum [57]. Research shows that CircHIPK3 [58] and erucic acid (SA) [59] can affect the occurrence of pyroptosis, thus protecting the systemic inflammatory response and intestinal mucosal barrier damage of AP [59]. SAP is also closely related to an increase in GSDMD levels. Activation of the GSDMD axis can stimulate pyroptosis, promote the release of inflammatory factors, induce intestinal injury, and downregulate the expression of the tight junction (TJ) protein markers ZO-1 and occludin, leading to intestinal mucosal damage. Downregulation of GSDMD protein reduces the intestinal damage related to SAP-related intestinal injury, reduces the degree of intestinal edema and bleeding, and alleviates the pathological changes in pancreatic tissue [60]. In addition, caspase-11 mediated pyroptosis is involved in SAP-induced AKI. Wedrolactone (Wed) is a caspase-11 inhibitor that can inhibit the IκB kinase (IKK) complex and inhibit the expression of caspase-11, which effectively inhibits the caspase-11-dependent pyroptosis signaling pathway and reduces mature IL-1β and GSDMD-N levels to protect against AP and AP-induced AKI [61,62]. Moreover, ALI is characterized by massive destruction of the pulmonary endothelial cell barrier, leading to pulmonary edema, inflow of proinflammatory leukocytes (especially neutrophils), and severe hypoxemia. In endotoxemia, high concentrations of endotoxin may persist and be abnormally located in the cytoplasm. Then, activated TLR4 induces the type I interferon response and complement C3–C3aR axis to upregulate the expression of caspase-11, trigger the overactivation of caspase-11, leading to high levels of pyroptosis and promoting the release of NETs, thus aggravating AP-related lung injury.[ 63,64].

### 3.5 Ferroptosis (Fig. 3)

Ferroptosis is a new RCD term created in 2012. At that time, researchers found that cell death induced by erastin and RAS selective lethal 3 (RSL3) was not affected by a caspase inhibitor (Z-VAD-FMK), necrostatin-1 and autophagy inhibitors (chloroquine, 3-methyl and adenine) [6,12]. Many serious and common human degenerative diseases, such as Parkinson's disease and Huntington's disease, and some acute injuries, such as stroke, cerebral hemorrhage, traumatic brain injury and ischemia‒reperfusion injury may be related to ferroptosis [65].Ferroptosis plays an important role in the death of acinar cells. Recent studies have shown that nucleoprotein 1 (NUPR1) transcription regulator 1-lipid transporter 2 (LCN2) can reduce iron-induced oxidative damage and induce l-arginine-induced ferroptosis in AP. LCN2 knockout mice were more prone to increased mortality, increased pancreatic tissue damage, increased serum amylase pancreatic myeloperoxidase (MPO, a marker of neutrophil recruitment), and increased serum HMGB1 [66,67]. Iron is closely related to AP damage. An animal study showed that a high iron diet or the conditional knockout of Gpx4 in the pancreas could promote the occurrence of experimental pancreatitis in mice, which was caused by taking carulein or l-arginine (a conditionally essential amino acid). In contrast, liproxstatin-1 (an iron prolapse inhibitor) reversed this type of pancreatitis injury, indicating the pathogenic role of iron prolapse in experimental pancreatitis [68,69]. Ferroptosis also plays a key role in SAP-related renal damage; inhibiting ferroptosis can improve SAP-related oxidative stress and renal dysfunction, which may be accompanied by a decrease in GPX4 activity and an increase in ferroptosis-related proteins and genes. GPX4 is a key gene for iron ptosis [70]. Nuclear factor NRF2 is a transcription factor that regulates the expression of antioxidant proteins and can prevent oxidative damage. Moreover, Nrf2 has been shown to protect cells from ferroptosis [71]. For example, BML-111, docosahexaenoic acid (DHA) and tanshinone IIA (TSA) have been shown to upregulate Nrf2 expression and activate its downstream HO-1/NQO-1 pathway to protect against cell death and tissue damage induced by ferroptosis, thereby reducing pancreatitis and intestinal epithelial damage [[72], [73], [74]]. Carbon monoxide (CO) is a downstream molecule of Nrf2 and CO donors based on nanotechnology, CO-bound hemoglobin vesicles (CO-HbVs), have been shown to inhibit neutrophil infiltration in the pancreas and reduce subsequent pancreatitis-related ALI [75]. Lastly, Wed inhibits oxidative stress and lipid peroxidation, upregulates the expression of GPX4, and inhibits ferroptosis to protect against AP and its related lung injury [61]. In addition, studies have shown that copper can promote ferroptotic cell death through GPX4-related autophagy pathways [76].

## 4 The connection between RCDs

**Fig. 4:**
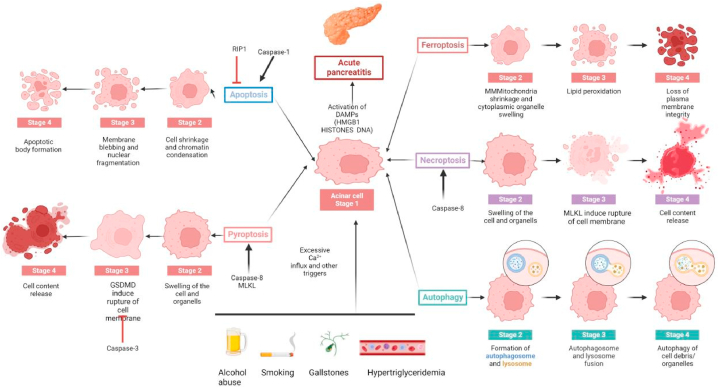
The connection between RCDs in acute pancreatitis [3,41,42].

Various death pathways are not completely isolated from each other and we believe that they are related. The occurrence and regulation of various cells in AP is also extremely complex. Preventing one type of RCD will induce the activation of another pathway [58]. Caspase-8 was initially identified to be involved in the occurrence of the extrinsic pathway. Soon, it became obvious that caspase-8 has a more complex role, especially in the regulation of other cell death pathways. Caspase-8 lethality can be rescued by deletion of RIP3 or MLKL, which indicates that caspase-8 restricts fecroptosis signaling. The enzymatic activity of caspase-8 determines whether cells survive or die via apoptosis or necroptosis [8,21]. Apoptosis-related caspase-3 cleaves pyroptosis-related GSDME, which is involved in the formation of mitochondrial pores and the enhancement of apoptotic signals [11]. Deficiency of Gpx4, a key regulator of ferroptosis, also showed sensitivity to apoptosis, necroptosis, and pyroptosis. Recent findings suggest that the induction of ferroptosis is associated with increased turnover of LC3 and the fusion of autophagosomes with lysosomes. The genetic deletion of autophagy-related proteins (Atg5 and Atg7) and other core autophagy molecules can block cell death through ferroptosis [12]. The molecular mechanism of autophagy leading to ferroptosis may involve several pathways, such as ferritin phage degradation via NCOA4-dependent ferritin (e.g., ferritin-specific autophagy) and inhibition of system X_C_^−^ activity by the formation of the Becn1-SLC7A11 protein complex [65]. Iron has been shown to induce oxidative stress by elevating ROS and links different RCDs including ferroptosis, apoptosis, pyroptosis and necroptosis. Ferroptosis and necroptosis have been implicated in kidney pathologies, and inhibitors of ferroptosis and necroptosis showed protection in various disease models in mice [6]. In addition to its role in apoptosis, AIFM2 was recently identified as a potent ferroptosis resistance factor (also called ferroptosis suppressor protein 1, FSP1), which functions as an oxidoreductase that reduces coenzyme Q10, generating a lipophilic radical-trapping antioxidant. FSP1 expression positively correlates with ferroptosis resistance across hundreds of cancer cell lines, and FSP1 mediates resistance to ferroptosis in lung cancer both in vivo and in vitro [25,77]. A shift in regulated cell death from apoptosis to pyroptosis as well as stimulation of necroptosis might explain why some of our patients deteriorated and developed a necrotizing course of pancreatitis [75,78]. These data highlight the importance of regulatory transitions between different forms of cell death that influence the onset and progression of acute pancreatitis [46] (see Fig. 4).

## 5 Conclusion

Acute pancreatitis is an inflammatory disease. It involves the escape of abnormally activated proteases and lipases from the acinus into the pancreatic stroma, leading to the autodigestion of the pancreatic tissue. It is often associated with SIRS and MOF. Current management guidelines for acute pancreatitis (AP) include intravenous rehydration, dietary changes, analgesics, pancreatic secretion inhibitors (somatostatin and its analog octreotide), l-arginine, calcium antagonists, and different inflammatory mediator inhibitors [5,6]. It has also been suggested that mesenchymal stem cells (MSCs), blood purification, chest ligation/drainage and other methods may play a positive role in the injury of SAP and related organs. However, a large amount of scientific research and clinical data are still needed to support and propose more effective targeted drugs.

## Funding

This work was supported by the 10.13039/501100001809National Natural Science Foundation of China (No.82260135).

## Author contribution statement

All authors listed have significantly contributed to the development and the writing of this article.

## Declaration of competing interest

The authors declare the following financial interests/personal relationships which may be considered as potential competing interests: All authors have no competing interests.

## References

[1] Lankisch P.G., Apte M., Banks P.A. (2015). Acute pancreatitis. Lancet.

[2] Boxhoorn L. (2020). Acute pancreatitis. Lancet.

[3] Mederos M.A., Reber H.A., Girgis M.D. (2021). Acute pancreatitis: a review. JAMA.

[4] Tarasiuk A., Fichna J. (2019). Effectiveness and therapeutic value of phytochemicals in acute pancreatitis: a review. Pancreatology.

[5] Lee P.J., Papachristou G.I. (2019). New insights into acute pancreatitis. Nat. Rev. Gastroenterol. Hepatol..

[6] Galluzzi L. (2018). Molecular mechanisms of cell death: recommendations of the nomenclature committee on cell death 2018. Cell Death Differ..

[7] Hoque R., Mehal W.Z. (2015). Inflammasomes in pancreatic physiology and disease. Am. J. Physiol. Gastrointest. Liver Physiol..

[8] Gaman L. (2018).

[9] Sendler M. (2020). NLRP3 inflammasome regulates development of systemic inflammatory response and compensatory anti-inflammatory response syndromes in mice with acute pancreatitis. Gastroenterology.

[10] Garg P.K., Singh V.P. (2019). Organ failure due to systemic injury in acute pancreatitis. Gastroenterology.

[11] Kist M., Vucic D. (2021). Cell death pathways: intricate connections and disease implications. EMBO J..

[12] Tang D. (2019). The molecular machinery of regulated cell death. Cell Res..

[13] Wang J. (2020). CircHIPK3 promotes pyroptosis in acinar cells through regulation of the miR-193a-5p/GSDMD Axis. Front. Med..

[14] Wang Q., Liu S., Han Z. (2020). miR-339-3p regulated acute pancreatitis induced by caerulein through targeting TNF receptor-associated factor 3 in AR42J cells. Open Life Sci..

[15] Xu X., Lai Y., Hua Z.C. (2019). Apoptosis and apoptotic body: disease message and therapeutic target potentials. Biosci. Rep..

[16] Bhatia M. (2004). Apoptosis of pancreatic acinar cells in acute pancreatitis: is it good or bad?. J. Cell Mol. Med..

[17] Mayerle J. (2019). Genetics, cell biology, and pathophysiology of pancreatitis. Gastroenterology.

[18] Kang R., Lotze M.T., Zeh H.J., Billiar T.R., Tang D. (2014). Cell death and DAMPs in acute pancreatitis. Mol. Med..

[19] Yang Y. (2020). MicroRNAs in acute pancreatitis: from pathogenesis to novel diagnosis and therapy. J. Cell. Physiol..

[20] Lv C., Jin Q. (2019). Maresin-1 inhibits oxidative stress and inflammation and promotes apoptosis in a mouse model of caerulein-induced acute pancreatitis. Med. Sci. Mon. Int. Med. J. Exp. Clin. Res..

[21] Najenson A.C., Courreges A.P., Perazzo J.C., Rubio M.F., Vatta M.S., Bianciotti L.G. (2018). Atrial natriuretic peptide reduces inflammation and enhances apoptosis in rat acute pancreatitis. Acta Physiol..

[22] Huang H., Swidnicka-Siergiejko A.K., Daniluk J., Gaiser S., Yao Y., Peng L. (2020). Transgenic expression of PRSS1(R122H) sensitizes mice to pancreatitis. Gastroenterology.

[23] Tan J.H., Cao R.C., Zhou L., Zhou Z.T., Chen H.J., Xu J. (2020). EMC6 regulates acinar apoptosis via APAF1 in acute and chronic pancreatitis. Cell Death Dis..

[24] Bhatia M. (2004). Apoptosis versus necrosis in acute pancreatitis. Am. J. Physiol. Gastrointest. Liver Physiol..

[25] Tan J.H., Cao R.C., Zhou L. (2020). ATF6 aggravates acinar cell apoptosis and injury by regulating p53/AIFM2 transcription in Severe Acute Pancreatitis. Theranostics.

[26] Abdelzaher W.Y., Ahmed S.M., Welson N.N., Marraiki N., Batiha G.E., Kamel M.Y. (2021). Vinpocetine ameliorates L-arginine induced acute pancreatitis via Sirt1/Nrf2/TNF pathway and inhibition of oxidative stress, inflammation, and apoptosis. Biomed. Pharmacother..

[27] Zhang D., Li L., Li J., Wei Y., Tang J., Man X. (2022). Colchicine improves severe acute pancreatitis-induced acute lung injury by suppressing inflammation, apoptosis and oxidative stress in rats. Biomed. Pharmacother..

[28] Bansod S., Godugu C. (2021). Nimbolide ameliorates pancreatic inflammation and apoptosis by modulating NF-kappaB/SIRT1 and apoptosis signaling in acute pancreatitis model. Int. Immunopharm..

[29] Klionsky D.J., Petroni G., Amaravadi R.K., Baehrecke E.H., Ballabio A., Boya P. (2021). Autophagy in major human diseases. EMBO J..

[30] Perez S., Pereda J., Sabater L., Sastre J. (2015). Redox signaling in acute pancreatitis. Redox Biol..

[31] Habtezion A., Gukovskaya A.S., Pandol S.J. (2019). Acute pancreatitis: a multifaceted set of organelle and cellular interactions. Gastroenterology.

[32] Mareninova O.A., Hermann K., French S.W., O'Konski M.S., Pandol S.J., Webster P. (2009). Impaired autophagic flux mediates acinar cell vacuole formation and trypsinogen activation in rodent models of acute pancreatitis. J. Clin. Invest..

[33] Saluja A., Dudeja V., Dawra R. (2019). Early intra-acinar events in pathogenesis of pancreatitis. Gastroenterology.

[34] Voronina S., Chvanov M., De Faveri F., Mayer U., Wileman T., Criddle D. (2022). Autophagy, acute pancreatitis and the metamorphoses of a trypsinogen-activating organelle. Cells.

[35] Vanasco V., Ropolo A., Grasso D. (2021). Mitochondrial dynamics and VMP1-related selective mitophagy in experimental acute pancreatitis. Front. Cell Dev. Biol..

[36] Zhu H., Huang L., Zhu S. (2016). Regulation of autophagy by systemic admission of microRNA-141 to target HMGB1 in l-arginine-induced acute pancreatitis in vivo. Pancreatology.

[37] Li H.Y., Lin Y.J., Zhang L., Zhao J., Xiao D.Y., Li P.W. (2021). Autophagy in intestinal injury caused by severe acute pancreatitis. Chin. Med. J..

[38] Wu Y., Tang L., Wang B., Sun Q., Zhao P., Li W. (2019). The role of autophagy in maintaining intestinal mucosal barrier. J. Cell. Physiol..

[39] Larabi A., Barnich N., Nguyen H.T.T. (2020). New insights into the interplay between autophagy, gut microbiota and inflammatory responses in IBD. Autophagy.

[40] Song G., Liu D., Geng X., Ma Z., Wang Y., Xie W. (2019). Bone marrow-derived mesenchymal stem cells alleviate severe acute pancreatitis-induced multiple-organ injury in rats via suppression of autophagy. Exp. Cell Res..

[41] Song G., Ma Z., Liu D., Zhou J., Meng H., Zhou B. (2019). Bone marrow-derived mesenchymal stem cells ameliorate severe acute pancreatitis by inhibiting necroptosis in rats. Mol. Cell. Biochem..

[42] Al Mamun A., Suchi S.A., Aziz M.A., Zaeem M., Munir F., Wu Y. (2022). Pyroptosis in acute pancreatitis and its therapeutic regulation. Apoptosis.

[43] Louhimo J., Steer M.L., Perides G. (2016). Necroptosis is an important severity determinant and potential therapeutic target in experimental severe pancreatitis. Cell Mol Gastroenterol Hepatol.

[44] Sun S., Han Y., Zhang C., Liu H., Wang B., Cao S. (2022). Adenosine kinase inhibition prevents severe acute pancreatitis via suppressing inflammation and acinar cell necroptosis. Front. Cell Dev. Biol..

[45] He R., Wang Z., Dong S., Chen Z., Zhou W. (2022). Understanding necroptosis in pancreatic diseases. Biomolecules.

[46] Sundar V., Senthil Kumar K.A., Manickam V. (2020). Current trends in pharmacological approaches for treatment and management of acute pancreatitis - a review. J. Pharm. Pharmacol..

[47] Boonchan M., Arimochi H., Otsuka K., Kobayashi T., Uehara H., Jaroonwitchawan T. (2021). Necroptosis protects against exacerbation of acute pancreatitis. Cell Death Dis..

[48] Yang X., Li R., Xu L., Qian F., Sun L. (2021). Serum amyloid A3 is required for caerulein-induced acute pancreatitis through induction of RIP3-dependent necroptosis. Immunol. Cell Biol..

[49] Cui Q.R., Ling Y.H., Wen S.H. (2019). Gut barrier dysfunction induced by aggressive fluid resuscitation in severe acute pancreatitis is alleviated by necroptosis inhibition in rats. Shock.

[50] Li H.Y., Lin Y.J., Zhang L., Zhao J., Xiao D.Y., Huang Z.Z. (2021). Progress of pyroptosis in acute pancreatitis. Chin. Med. J..

[51] Wang J., Wang L., Zhang X., Xu Y., Chen L., Zhang W. (2021). Cathepsin B aggravates acute pancreatitis by activating the NLRP3 inflammasome and promoting the caspase-1-induced pyroptosis. Int. Immunopharm..

[52] Hoque R., Sohail M., Malik A., Sarwar S., Luo Y., Shah A. (2011). TLR9 and the NLRP3 inflammasome link acinar cell death with inflammation in acute pancreatitis. Gastroenterology.

[53] Heilig R., Dick M.S., Sborgi L., Meunier E., Hiller S., Broz P. (2018). The Gasdermin-D pore acts as a conduit for IL-1beta secretion in mice. Eur. J. Immunol..

[54] Rau B., Paszkowski A., Lillich S. (2001 Jul). Differential effects of caspase-1/interleukin-1beta-converting enzyme on acinar cell necrosis and apoptosis in severe acute experimental pancreatitis. Lab. Invest..

[55] Ye A., Li W., Zhou L., Ao L., Fang W., Li Y. (2020). Targeting pyroptosis to regulate ischemic stroke injury: molecular mechanisms and preclinical evidences. Brain Res. Bull..

[56] Hoque R., Mehal W.Z. (2015). Inflammasomes in pancreatic physiology and disease. Am. J. Physiol. Gastrointest. Liver Physiol..

[57] Zhang X.H., Li M.L., Wang B., Guo M.X., Zhu R.M. (2014). Caspase-1 inhibition alleviates acute renal injury in rats with severe acute pancreatitis. World J. Gastroenterol..

[58] Wang J., Li X., Liu Y., Peng C., Zhu H., Tu G. (2020). CircHIPK3 promotes pyroptosis in acinar cells through regulation of the miR-193a-5p/GSDMD Axis. Front. Med..

[59] Huang Z.W., Tan P., Yi X.K., Chen H., Sun B., Shi H. (2022). Sinapic acid alleviates acute pancreatitis in association with attenuation of inflammation, pyroptosis, and the AMPK/NF-KB signaling pathway. Am. J. Chin. Med..

[60] Wu J., Zhang J., Zhao J., Chen S., Zhou T., Xu J. (2021). Treatment of severe acute pancreatitis and related lung injury by targeting gasdermin D-mediated pyroptosis. Front. Cell Dev. Biol..

[61] Fan R., Sui J., Dong X., Jing B., Gao Z. (2021). Wedelolactone alleviates acute pancreatitis and associated lung injury via GPX4 mediated suppression of pyroptosis and ferroptosis. Free Radic. Biol. Med..

[62] Shao Y., Li C., Jiang Y., Li H., Tang X., Gao Z. (2023). Inhibition of caspase-11-mediated pyroptosis alleviates acute kidney injury associated with severe acute pancreatitis in rats. J. Invest. Surg..

[63] Oh C., Verma A., Aachoui Y. (2020). Caspase-11 non-canonical inflammasomes in the lung. Front. Immunol..

[64] Wu X.B., Sun H.Y., Luo Z.L., Cheng L., Duan X.M., Ren J.D. (2020). Plasma-derived exosomes contribute to pancreatitis-associated lung injury by triggering NLRP3-dependent pyroptosis in alveolar macrophages. Biochim. Biophys. Acta, Mol. Basis Dis..

[65] Yuan H., Pratte J., Giardina C. (2021). Ferroptosis and its potential as a therapeutic target. Biochem. Pharmacol..

[66] Liu J., Song X., Kuang F. (2021). NUPR1 is a critical repressor of ferroptosis. Nat. Commun..

[67] Martin T.A. (2021). *NUPR1 and its potential role in cancer and pathological conditions (Review)*. Int J Oncol.

[68] Kimita W., Petrov M.S. (2020). Iron metabolism and the exocrine pancreas. Clin. Chim. Acta.

[69] Chand S.K., Singh R.G., Pendharkar S.A. (2018). Interplay between innate immunity and iron metabolism after acute pancreatitis. Cytokine.

[70] Ma D., Li C., Jiang P. (2021). Inhibition of ferroptosis attenuates acute kidney injury in rats with severe acute pancreatitis. Dig. Dis. Sci..

[71] Li H., Lin Y., Zhang L., Zhao J. (2022). Ferroptosis and its emerging roles in acute pancreatitis. Chin. Med. J..

[72] Xue Q. (2023). Copper-dependent autophagic degradation of GPX4 drives ferroptosis. Autophagy.

[73] Shi Z., Wang Y., Ye W. (2021). The LipoxinA4 receptor agonist BML-111 ameliorates intestinal disruption following acute pancreatitis through the Nrf2-regulated antioxidant pathway. Free Radic. Biol. Med..

[74] Ahn Y.J., Lim J.W., Kim H. (2020). Docosahexaenoic acid induces expression of NAD(P)H: quinone oxidoreductase and heme oxygenase-1 through activation of Nrf2 in cerulein-stimulated pancreatic acinar cells. Antioxidants.

[75] Chen W., Yuan C., Lu Y. (2020). Tanshinone IIA protects against acute pancreatitis in mice by inhibiting oxidative stress via the Nrf2/ROS pathway. Oxid. Med. Cell. Longev..

[76] Taguchi K., Nagao S., Maeda H. (2018). Biomimetic carbon monoxide delivery based on hemoglobin vesicles ameliorates acute pancreatitis in mice via the regulation of macrophage and neutrophil activity. Drug Deliv..

[77] Mayerle J., Sendler M., Hegyi E., Beyer G., Lerch M.M., Sahin-Toth M. (2019). Genetics, cell biology, and pathophysiology of pancreatitis. Gastroenterology.

[78] Sendler M., van den Brandt C., Glaubitz J. (2020). NLRP3 inflammasome regulates development of systemic inflammatory response and compensatory anti-inflammatory response syndromes in mice with acute pancreatitis. Gastroenterology.

